# The Immune Landscape of Acral Melanoma: From Basic to Clinical

**DOI:** 10.1002/cam4.71615

**Published:** 2026-05-04

**Authors:** Lihong Jiang, Zhaotian Zhang

**Affiliations:** ^1^ Cancer Institute (Key Laboratory of Cancer Prevention and Intervention, China National Ministry of Education), The Second Affiliated Hospital, School of Medicine, Zhejiang University Hangzhou Zhejiang China; ^2^ State Key Laboratory of Transvascular Implantation Devices Hangzhou China

**Keywords:** acral melanoma, combination regimen, drug resistance, immune checkpoint, immunotherapy

## Abstract

**Background:**

Acral melanoma (AM) is an aggressive melanoma subtype with poor prognosis and limited response to immune checkpoint inhibitors (ICIs). Despite increasing research efforts, the mechanisms underlying therapeutic resistance remain incompletely understood.

**Aim:**

This review examines the mechanisms driving immunotherapy resistance in AM, summarizes current clinical advances in combination regimens, and explores future therapeutic directions.

**Methods:**

A narrative review of recent literature was undertaken, encompassing studies on resistance mechanisms and clinical trials investigating novel ICI‐based combination therapies for AM.

**Results:**

AM exhibits distinct immunosuppressive microenvironment characterized by low tumor mutational burden, reduced CD8+ T‐cell infiltration, enrichment of regulatory T cells, and specific genetic alterations. Emerging clinical data demonstrate that combination regimens—particularly dual ICIs (anti‐PD‐1 plus anti‐CTLA‐4) and ICI combinations with anti‐angiogenic agents or chemotherapy—have shown promising efficacy, with some achieving superior response rates in AM patients.

**Discussion:**

Understanding resistance mechanisms is critical for identifying novel therapeutic targets and optimizing personalized strategies. Current evidence suggests combination therapies may overcome resistance and improve outcomes, though optimal regimens and sequencing require further investigation.

**Conclusion:**

Continued research into innovative combination approaches and predictive biomarkers is urgently needed to improve survival in AM.

**Abbreviations**
β‐AR: β‐adrenergic receptor
ACT: adoptive cell transfer
AM: acral melanoma
ANG2: angiopoietin‐2
APCs: antigen‐presenting cells
CAR‐T: chimeric antigen receptor T cell
CM: cutaneous melanoma
CNAs: copy number abnormalities
CRS: cytokine release syndrome
DOR: duration of response
FMT: fecal microbiome transplantation
ICB: immune checkpoints blockade
ICIs: immune checkpoint inhibitors
IDO1: indoleamine 23‐dioxygenase 1
IFN: interferon
irAEs: immune‐related adverse events
mAb: monoclonal antibody
MDSCs: marrow‐derived suppressor cells
MM: mucosal melanoma
NAM: nail apparatus melanoma
NK: natural killer
NLR: neutrophil‐to‐lymphocyte ratio
NM: nodular melanoma
ORR: objective response rate
OS: overall survival
pCR: pathological response rates
PD: progressive disease
pDCs: plasmacytoid dendritic cells
PFS: progression‐free survival
PSM: palm and sole melanoma
RFS: recurrence‐free survival
SNPs: single nucleotide sequence changes
STING: stimulator of interferon genes
TAMs: tumor‐associated macrophages
TCR: T cell receptor
Tex: exhausted T cells
TGF‐β: transforming growth factor‐beta
TILs: tumor‐infiltrating lymphocytes
TLR9: toll‐like receptor 9
TMB: tumor mutation burden
TME: tumor microenvironment
TMZ: temozolomide
Tregs: regulatory T cells
T‐VEC: talimogene laherparepvec
TWT: triple wild type
UV: ultraviolet
VEGF: vascular endothelial growth factor

## 1 Introduction

Acral melanoma (AM) is a subtype of melanoma and constitutes 2%–3% of melanomas [1, 2]. AM witnessed an annual increase of approximately 1.7% in incidence rates and had a higher morbidity among individuals with darker skin, posing significant threats to human health [3]. Different from the high prevalence of cutaneous melanoma (CM) in western countries, AM predominantly affects less developed regions such as Asia, Africa, and Latin America, resulting in high mortality rates. Moreover, AM exhibited a higher degree of invasiveness compared to CM, which was associated with worse prognosis [4]. The 5‐year survival rate of AM was lower than that for malignant CM overall (80.3% vs. 91.3%), which was only 29% in Japanese and this type served as an independent poor prognostic factor in stage IIIA [4, 5].

AM is commonly found in the soles of the feet, palms, and under nails, which are areas lacking hair follicles and experiencing reduced exposure to ultraviolet (UV) [6]. The relationship between AM and UV remains inconspicuous; however, showing the potential associations with physical stress, trauma, or persistent inflammation [7]. In addition, older age, lower socioeconomic status, and concurrent presence or history of other tumors are also reported to be related to the occurrence of AM [8]. Histologically, AM is characterized by the presence of solitary or nested proliferation of atypical melanocytes and an infiltrative growth pattern at the dermal‐epidermal junction. Areas of lymphocytic infiltration and thickening of the surrounding epidermis are commonly observed in AM [9].

Currently, AM represents a challenging form of skin cancer characterized by rapid progression, delayed diagnosis, and unfavorable prognosis. The lack of public awareness regarding AM, limited availability of early diagnostic methods, and the concealed location of certain tumors contribute to the inherent difficulty in accurately diagnosing this disease [10]. Meanwhile, existing chemotherapy, targeted therapy, and immunotherapy approaches available for progressive CM have limited efficacy in AM patients, which is mainly embodied in the significantly shorter survival in AM. Therefore, there is an urgent need for further basic and translational research to address these challenges. This review article encompasses an analysis of recent clinical trial data pertaining to immunotherapy for AM, focuses on elucidating the resistance mechanisms associated with immunotherapy for this condition, conducts a thorough evaluation of efficacy and safety profiles of current combination therapy, and explores the potential future directions for AM systemic therapy.

## 2 Immunotherapy in AM Management

### 2.1 Tumor Microenvironment (TME) of AM

TME comprises cancer cells, neighboring cells (immune cells, fibroblasts, bone marrow‐derived inflammatory cells), blood vessels and lymphatics, diverse cellular molecules, and extracellular matrix. The progression of cancer is associated with an interplay between cancer cells and their microenvironment akin to the relationship between ‘seed’ and ‘soil’, namely TME exerts a pivotal influence on the malignant advancement of tumors, immune evasion, and the emergence of drug resistance [11]. With melanoma, tumor cells secrete inhibitory cytokines to facilitate the establishment of an immunosuppressive TME, which leads to enhanced antiapoptotic capabilities of melanoma cells, simultaneously gradually exhausted immune cells with decreased cytotoxicity and cytokine production. Additionally, there is an upregulation of immunosuppressive receptors such as PD‐1, PD‐L1, CTLA‐4, and TIM‐3 on the immune cell surface inhibiting their activity or inducing apoptosis [12].

Compared to CM, AM was reported to exhibit more pronounced immunosuppression, characterized by lower immune infiltration with significant reductions in effector CD8+ T cells, natural killer (NK) cells, plasmacytoid dendritic cells (pDCs) and γδT cells, enrichment of regulatory T cells (Tregs) and exhausted T cells (Tex) frequently coexist with higher levels of inhibitory markers such as PD‐1, TIM‐3, LAG‐3, VISTA, TIGIT, ADORA2 and TNFRSF9 [13, 14]. Several studies reported different markers representing the exhausted state of T or NK cells in the TME of AM. The expression of PD‐1 and TIM‐3 in Tex was found to be markedly increased while higher expression of CTLA‐4 and LAYN was seen in Tregs [15]. Besides lymphocytes, some myeloid cells such as cancer‐associated fibroblasts (CAFs) that specifically express CTGF were also reported to drive an immunosuppressive TME [15]. Additionally, the immune microenvironment in AM also displayed features such as an elevated neutrophil‐to‐lymphocyte ratio (NLR), which are typically associated with disease progression and poor prognosis in patients.

**FIGURE 1:**
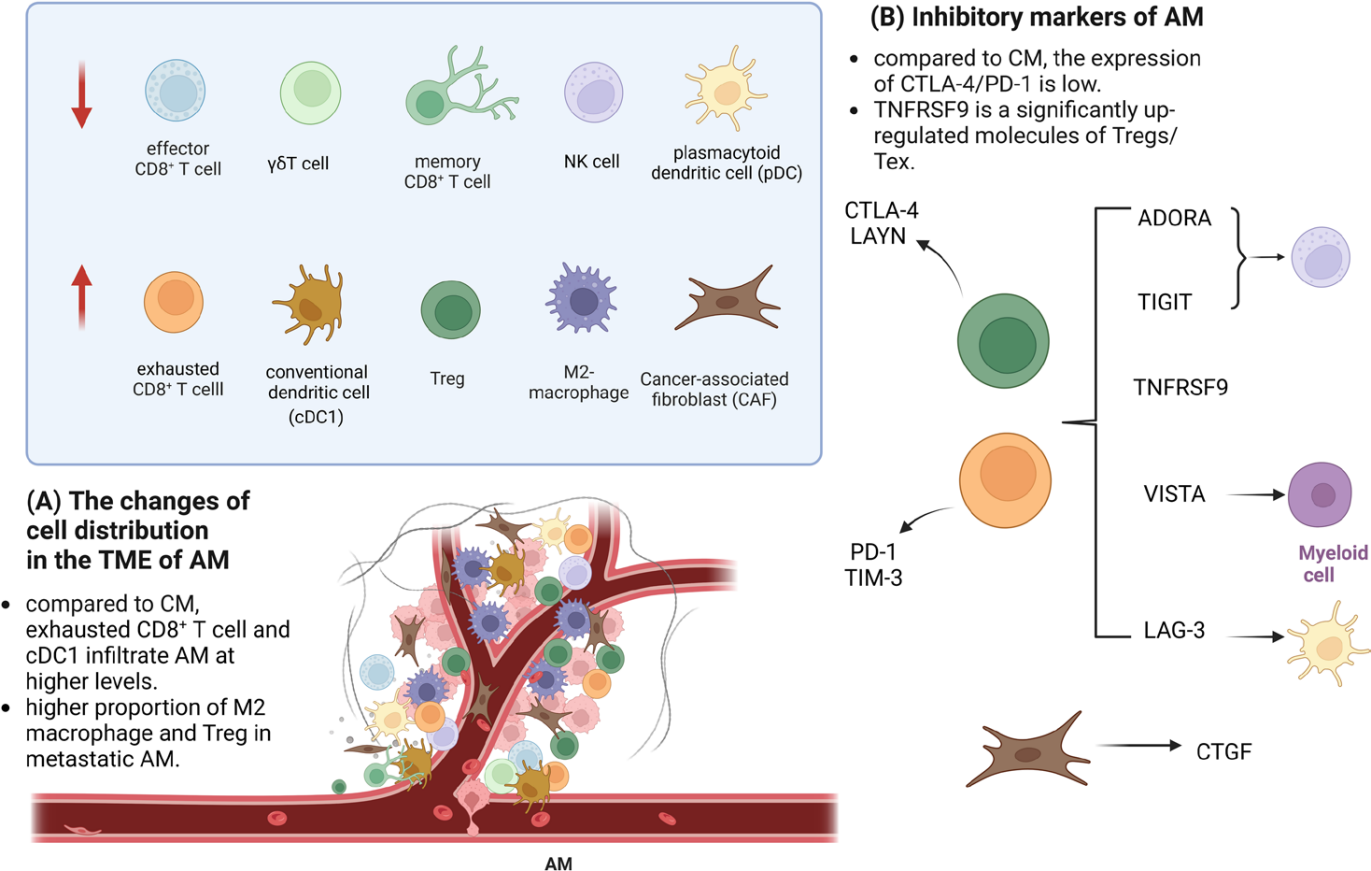
Characteristics of TME in AM. (A) AM exhibits a more immunosuppressive TME than that of CM, characterized by lower immune infiltration in immune cells with effector and cytotoxic or antigen‐presenting functions, enrichment of exhausted or inhibitory immune cells. (B) Within the TME of AM, cells express higher levels of inhibitory markers. CTLA‐4 and LAYN are highly expressed in the Tregs while exhausted T cells show higher expression of PD‐1 and TIM‐3. Additionally, immune checkpoints including LAG‐3, VISTA, TIGIT, ADORA2, and TNFRSF9 are also highly expressed in the immune cells, which may coexist in various cells infiltrating in the tumors, including some myeloid cells. This figure was created with BioRender.com. AM, acral melanoma; CAFs, cancer‐associated fibroblasts; cDCs, conventional dendritic cells; CM, cutaneous melanoma; pDCs, plasmacytoid dendritic cells; Tregs, regulatory T cells; TME, tumor microenvironment.

Due to these distinct features of the immune microenvironment, prognosis and treatment response can vary significantly among AM patients. For instance, an abundance of M1‐macrophages within the stromal region generally suggests a favorable prognosis for AM [16]. Another research study found that resistance to immunotherapy in an AM patient with brain metastases was associated with a higher proportion of M2‐macrophages and Tregs in the brain TME compared to the primary skin lesion [17]. In summary, AM exhibits a diverse and complex TME. The accurate identification of environmental characteristics is essential for assessing patient prognosis and treatment response while providing a basis for selecting effective treatments. The characteristics of the TME in AM are shown in Figure 1.

### 2.2 Immune Checkpoint Inhibitors (ICIs) Applied in AM

Immune checkpoints, a class of molecules that regulate immune activation, typically express on the surface of immune cells. Under normal circumstances, they play a critical role in modulating excessive immune responses to safeguard tissues from damage. However, cancer cells often activate this regulatory system by upregulating the expression of their ligands, thereby facilitating cancer progression. The fundamental principle underlying ICIs is to restore the functionality of immune cells by specifically targeting receptors or ligands, ultimately reactivating antitumor effects. Currently, anti‐PD‐1 monoclonal antibody (mAb) such as nivolumab, pembrolizumab and anti‐CTLA‐4 mAb such as ipilimumab are predominantly utilized in the treatment of advanced melanoma including AM [18]. Relevant studies have demonstrated that the objective response rate (ORR), overall survival (OS) and progression‐free survival (PFS) of AM patients treated with nivolumab or pembrolizumab were comparable to the data published in CM patients. Moreover, blocking PD‐1/PD‐L1 and CTLA‐4 concurrently could significantly prolong the OS in advanced AM patients, superior to either agent used individually [19]. However, the application efficacy of ICIs in AM remains suboptimal, and several challenges persist. Due to the unique characteristics of immune TME in AM, the ORR of immunotherapy is often lower than that of CM in most retrospective studies [19]. Moreover, ICIs contribute to the higher rates of drug resistance in AM. Among patients with unresectable AM, although the ORR was numerically higher in the anti‐PD‐1/ipilimumab combination group, this superiority did not turn into increased OS [20]. Therefore, it is crucial to find novel targets for immune checkpoint blockade or synergistically combine ICIs with other treatment modalities based on the TME characteristics of AM to effectively counteract cancer cells' evasion from immune surveillance.

### 2.3 Other Immunotherapy Applications in AM

#### 2.3.1 Non‐Classical Immune Checkpoint Inhibitors

Based on a comprehensive investigation into the TME characteristics of AM, several novel immunotherapy targets have been identified with translational significance, including LAG‐3, TIM‐3, VISTA, TIGIT, and ADORA2 [13, 14]. VISTA is encoded by the VSIR gene as a transmembrane protein that shares similarities with PD‐L1 in its extracellular domain (ECD) and primarily exhibits the expression in lymphoid organs and bone marrow cells. As for TME, up‐regulation of VISTA occurs significantly within suppressor immune cells to inhibit CD4+ or CD8 + T cell proliferation while promoting the transformation of naive T cells into Tregs [21]. Furthermore, there is a gradual increase in VISTA expression during the progression of melanoma, suggesting its potential utility as a molecular marker for cancer prognosis [22]. TIM‐3, also known as HAVCR2, is a type 1 transmembrane protein that negatively regulates T cell responses in chronic viral infections and malignancies. It exhibits predominant expression on Th1, Tc1 cells, suppresses the proliferation of T cells and promotes their apoptosis. TIM‐3 is also expressed on NK cells and Tregs [23]. Studies have demonstrated a negative correlation between TIM‐3 expression levels and progression/survival time for patients with melanoma, and coexpression of TIM‐3 with PD‐L1 on T cells indicates a more exhausted state of these cells [13]. Moreover, elevated TIM‐3 expression was observed in AM patients resistant to monotherapy compared to those respond well to treatment [24]. LAG‐3 is constitutively expressed on Tregs, γδT cells, NK cells, pDCs, and activated B cells, as well as upregulated on the surface of CD4 + T and CD8 + T cells. In addition to inhibiting signal transduction following T cell activation, LAG‐3 was found to promote the secretion of various immunosuppressive cytokines and impede the proliferation of NK cells and pDCs [25]. Similarly, elevated levels of LAG‐3 or the simultaneous expression of PD‐1 and LAG‐3 in AM were found to be frequently associated with an unfavorable prognosis for patients and resistance to immunotherapy [26]. Currently, there are many drugs targeting these new immune checkpoints under investigation, such as relatlimab (anti‐LAG‐3), LY3321367 (anti‐TIM‐3) and CA‐170 (anti‐VISTA), that are being evaluated for melanoma treatment in clinical trials and some have demonstrated to be more effective than classical ICIs [27].

Despite the existing studies demonstrating up‐regulation of VISTA, TIM‐3, LAG‐3, and other molecules in the TME of AM, the precise mechanisms governing their regulation of intercellular interactions as well as their expression changes and functions in diverse immune processes remain elusive. In addition, there is little research conducted for these immune checkpoints in AM and the influence of new ICIs for immune resistance patients remains to be uncovered. Therefore, further investigation is warranted to elucidate specific roles of these molecules playing in AM.

#### 2.3.2 Adoptive Cell Therapy

Tumor‐infiltrating lymphocytes (TILs) therapy is a form of adoptive cell therapy, wherein TILs derived from tumor tissue are cultured and expanded in vitro on a large scale, then TILs possessing tumor‐killing capabilities are reintroduced into the patient to directly eliminate the tumor or enhance immune response. Hirai I et al. found that cancer‐reactive TILs can be expanded in a less immunosuppressive environment of melanoma [28]. AM patients generally have lower levels of TILs, which is associated with a poor prognosis [29]. Lifileucel is the first FDA‐approved TIL therapy for solid tumors, offering new hope for patients with advanced melanoma, particularly those who have failed multiple prior therapies [30]. Based on recent clinical trial results, TILs therapy has demonstrated greater effectiveness in prolonging PFS and OS for patients with AM, while shown lower toxicity and manageable duration, indicating its potential as an effective treatment [31].

Another cellular therapy known as CAR T (chimeric antigen receptor T cell) therapy, involves transferring specific genetic materials containing antigen recognition domains and T cell activation signals into modified T cells, enabling direct activation of these engineered T cells upon binding to cancer‐specific antigens to directly kill cancer cells. Current major therapeutic targets for melanoma include CD20, CD126, CD70, B7H3, VEGFR‐2, and gp100/HLA‐A2 complex GD2 [32]. Notably, studies found GD2 was highly expressed in melanoma of Chinese patients, especially AM patients (50%), providing a clinically appealing treatment strategy for these patients. A phase I clinical trial investigating GD2‐CAR‐T‐cell‐based therapy for melanoma is currently underway [33].

#### 2.3.3 Oncolytic Viruses

Oncolytic viruses represent a novel modality of immunotherapy that enhances efficacy by inducing tumor‐specific T cell responses. Viola Franke et al. administered the lytic tumor phage Talimogene laherparepvec (T‐VEC) to an AM patient and achieved a successful complete histologic response [34]. Moreover, after a 3‐year treatment with OrienX010 (an oncolytic virus), favorable antitumor effects were also observed in unresectable AM in both injected and non‐injected metastatic lesions [35]. Currently, oncolytic viruses have demonstrated synergistic effects when combined with treatments such as ICIs, radiotherapy, and chemotherapy, thereby holding promise in the future.

## 3 Resistance to Immunotherapy

### 3.1 The Drug Resistance in AM

The widespread utilization of ICIs has led to a significant improvement in the survival rate of patients with advanced melanoma in recent years. However, the primary resistance of melanoma is observed in approximately 40%–45% of patients treated with anti‐PD‐1 mAb and around 80% of patients with anti‐CTLA‐4 mAb, respectively. Furthermore, some patients initially exhibiting a favorable clinical response to ICIs experience tumor progression or recurrence after a certain period of treatment [36]. It's noteworthy that patients with AM tend to exhibit higher rates of immunotherapy resistance [37]. To develop an effective therapy, the foremost priority lies in elucidating the mechanisms underlying primary and acquired resistance to ICIs in AM while exploring strategies aimed at overcoming the immune resistance to guide future systemic therapy.

### 3.2 Potential Mechanisms of Immune Resistance in AM

#### 3.2.1 Activation of Inhibitory Immune Checkpoints

Immunosuppressive ligands expressed on the surface of cancer cells, such as PD‐L1/L2, effectively inhibit antitumor responses by interacting with immune cell surface receptors. There is generally a positive correlation between PD‐L1 expression and response to anti‐PD‐1/PD‐L1 mAb. The down‐regulation of PD‐L1 expression and up‐regulation of other immune checkpoints in tumor cells are believed to be important factors contributing to resistance against ICIs treatment [38].

Epigenetic regulation is the reversible modification of DNA and histones that controls gene expression without altering the DNA sequence. The expression of PD‐L1 in melanoma cells is regulated by various modifications, directly impacting the efficacy of ICIs [39]. For example, PD‐L1 expression was induced by IFN‐γ through the JAK/STAT1/IRF pathway while HDAC inhibitors facilitated PD‐L1 expression by enhancing histone acetylation upstream of the PD‐L1 gene [40]. As for transcriptional factors, loss of ATF3 enhanced the effectiveness of ADORA1 antagonists in inhibiting melanoma cell growth, while nuclear factor E2‐related transcription factor 2 (NRF2) promoted immune evasion of melanoma cells through the upregulation of PD‐L1 [41]. In terms of posttranslational modifications (PTMs), chemokine‐like factor superfamily member 6 (CMTM6) prevented ubiquitin‐mediated degradation of PD‐L1, while the upregulation of E3 ligase activity induced by caspase‐8 led to PD‐L1 degradation in melanoma cells [42]. Moreover, studies have shown that melanoma patients who showed a poor reaction to ICIs treatment displayed elevated levels of KDM5B (a histone H3K demethylase) or increased activity of EZH2 (a histone methyltransferase) [43]. These results highlight epigenetic modifiers that regulate the expression of PD‐L1 can consequently change the reaction of melanoma cells to ICIs, providing a reference for strategies to overcome immune resistance. AM exhibits a lower level of PD‐L1 expression, which contributes to poor prognosis and limited response to immunotherapy. Thus, increasing tumor‐specific PD‐L1 expression by epigenetic modifiers represents a promising strategy [14]. However, the specific impact of combining these drugs with immunotherapy on drug resistance in AM remains largely unknown and needs to put more effort into it for developing innovative therapies.

The inhibition of PD‐1 or CTLA‐4 can potentially trigger the activation of alternative immunoregulatory pathways within TILs, and these alterations promote therapeutic resistance. Higher expression of TIM‐3 within PD‐1^(+)^ CD8+ T cells compared to PD‐1^(−)^ CD8+ T cells was found in AM, which is associated with enhanced functional impairment and cellular exhaustion [44]. Furthermore, numerous studies have demonstrated an upregulation of VISTA, TIGIT, LAG‐3, and other immune cell surface molecules in anti‐PD‐1‐resistant melanoma. Therefore, targeting these molecules may hold the potential in augmenting the antitumor efficacy of immune cells in melanoma. Nevertheless, with more inhibitory molecules existing in the TME of AM, concurrent inhibition of multiple pathways may synergistically alleviate tumor‐induced suppression to achieve durable tumor remission.

#### 3.2.2 Intrinsic Factors of Tumor

Tumor mutation burden (TMB) and tumor immunogenicity play a crucial role in determining the response of tumors to immunotherapy. TMB quantifies the number of mutations per million DNA bases, serving as an indicator for genomic variability within tumor cells. Multiple studies consistently demonstrate that AM exhibits lower levels of TMB, indicating reduced expression of neoantigens and consequently a higher likelihood of evading host antitumor responses [45]. Conversely, high TMB has been identified as a positive prognostic factor for ICIs treatment in AM patients, as well as those exhibiting low structural variation typically achieving superior outcomes in terms of PFS. However, another study found TMB and chromosomal structural mutations uncorrelated with antitumor immunity in AM [46].

Human leukocyte antigens class I (HLA‐I) molecules expressed on the surface of nucleated cells are responsible for presenting endogenous antigens to the cell surface, which are recognized by CD8+ T cells and subsequently activated to eliminate corresponding cells. In cases where cancer cells lack HLA‐I molecules, antigen presentation becomes impaired, resulting in immune evasion by tumors. B2M is crucial for proper folding and trafficking of HLA‐I molecules; when the B2M gene is deactivated or mutated, lack of HLA‐I molecules results in the acquired resistance to anti‐PD‐1 mAb in patients with melanoma [47]. However, Kageshita T et al. discovered AM expressed a level of HLA‐I antigens similar to that of nodular melanoma (NM), but displayed a markedly lower expression than NM of the membrane bound high molecular weight melanoma associated antigen (HMW‐MAA), indicating a potential role of HMW‐MAA rather than HLA‐I in the development of immune resistance in AM [48].

The alterations of signaling pathways that disrupt immune response are critical factors in the development of drug resistance in tumors. BRAF inhibitors were found to increase the presence of tumor‐infiltrating T cells in melanoma xenograft models, indicating that some cytokines produced by the activation of the MAPK pathway can hinder the recruitment of T cells [49]. Likewise, the WNT/β‐catenin pathway induced immune resistance by reducing immune cell infiltration. Melanoma cells exhibiting high β‐catenin expression inhibit CCL4 expression and subsequently hamper CD103+ DCs recruitment, ultimately affecting T cell infiltration. However, among immune‐resistant AM patients, there was a decrease in the mRNA signatures associated with the WNT and IFNA1 signaling during treatment [45]. Moreover, the PI3K‐AKT pathway activation accompanied by PTEN loss is associated with diminished infiltration of intra‐tumoral T cells, as well as increased inhibitory cytokines contributing to a poor response to anti‐PD‐1 mAb [45, 50]. Melanoma cells lacking the IFN‐γ pathway exhibit reduced susceptibility to T cell‐mediated attack, either [51]. Nevertheless, the activation of the IFN‐γ pathway has a dual effect on the antitumor immune response influenced by genetic and transcriptomic changes in melanoma cells chronically exposed to IFN‐γ. Resistance to anti‐CTLA‐4/PD‐1 mAb often coincides with genetic alterations in the IFN‐γ pathway, such as the copy number loss of IFNGR1, JAK1, and JAK2, as well as the amplification of SOCS1 and PIAS4 [52]. Yu, J. et al. found a significantly increased expression of PD‐L1 was observed in some AM patients treated with CDK4 inhibitors, and further research showed CDK4 could facilitate PD‐L1 ubiquitination through the SPOP/Cullin 3‐SPOP E3 ligase [53]. These findings provide a theoretical basis for combining CDK4/6 inhibitors with anti‐PD‐1/L1 mAb against AM.

#### 3.2.3 Local Immunosuppressive TME

In the adaptive immune response against melanoma, tumor‐infiltrating T cells, particularly CD8+ T cells, are considered a favorable prognostic feature for malignant melanoma; however, individuals with primary resistance to melanoma often exhibit a deficiency in these cells. To impede T cell migration across the endothelial barrier into the tumor, vascular endothelial growth factor (VEGF) plays a pivotal role [54]. Furthermore, inhibition of VEGF results in the upregulation of angiopoietin‐2 (ANG2), which consistently promotes aberrant tumor angiogenesis, thereby counteracting the effects of anti‐VEGF therapy. Consequently, targeting both VEGF and ANG2 emerges as a crucial strategy for restoring T‐cell infiltration [55].

T cell exhaustion refers to the functional impairment of T cell receptor (TCR) due to persistent antigen stimulation, and it also plays a crucial role in facilitating acquired resistance to immunotherapy. This phenomenon features reduced production of effector cytokines by T cells, accompanied by increased expression of inhibitory receptors or immunosuppressive enzymes [56]. In vitro studies have demonstrated that the resistant mechanism to anti‐PD‐1 mAb may be attributed to the upregulation of PD‐1^high^ subset; however, the beneficial response to treatment can only be achieved by utilizing PD‐1^low^ cells that are not completely depleted [57]. Moreover, the presence of alternative exhausted subgroups may result in diminished efficacy of current immune checkpoints blockade (ICB) therapy. For instance, a highly activated subset of CD39+ T cells that existed in both blood and tumors of melanoma patients is strongly associated with the ICB resistance. Notably, the exhausted state of immune cells tends to be more pronounced in AM, which may explain why AM exhibits a higher resistance rate. Saraí G De et al. discovered conventional type 1 dendritic cells (cDC1) and exhausted CD8+ T cells, the most relevant immune cell types for antitumor responses, infiltrate in AM at similar or higher levels than CM [58].

Immunoregulatory cells are a group of cells from different types with the same features as inhibiting antitumor activity of immune cells, which are closely associated with resistance to ICB. In cases of melanoma drug resistance, TME typically recruits and expands immunoregulatory cells, such as Tregs, bone marrow‐derived suppressor cells (MDSCs), and tumor‐associated macrophages (TAMs). Tregs, a key to the immune suppressive TME in solid tumors, are frequently accumulated in melanoma and exert different immune effects on various immune cells within the tumor. Importantly, Tregs can release cytokines such as IL‐10 and TGF‐β to directly inhibit effector T cell response. In addition, HLA class II melanoma cells may directly activate and expand Tregs, which promotes the immune evasion of tumor cells [59]. Compared to CM, AM exhibits a higher infiltration rate of Tregs with increased expression of inhibitory markers including TIGIT, CTLA4, and LAYN [13]. As a diverse population of immature cells originating from bone marrow, MDSCs exhibit significant expansion in the peripheral blood and tumor tissues of melanoma patients. Apart from facilitating neovascularization and tumor cell infiltration to drive tumor progression, MDSCs also possess immunosuppressive properties by inhibiting T cells, NK cells, and dendritic cells (DCs) activities while promoting Tregs proliferation [60]. Furthermore, the presence of melanoma‐infiltrating MDSCs, as well as an increased proportion of MDSCs (CD33 + CD11b + HLA‐DR‐) in peripheral blood is inversely associated with the effectiveness of ICB. These findings suggest that the frequency and phenotype of MDSCs can serve as potential markers to predict the response rates and survival outcomes among AM patients undergoing ICB therapy [61].

TAMs are a special group of macrophages existing in TME, which have an indispensable influence on the efficacy of immunotherapy. Due to the predominant characteristics of hypoxia and weak acidity in the TME, most TAMs exhibit an M2 phenotype that weakens antitumor immunity, which contributes to inducing and sustaining an immunosuppressive TME through secretion of inhibitory cytokines, as well as expression of immune checkpoints and abnormal amino acid metabolism [62]. Conversely, the presence of M1 macrophages is associated with a favorable prognosis in AM patients, whereas a nivolumab‐resistant TME is characterized by an increased proportion of M2 macrophages [16]. Furthermore, inhibiting polarization of macrophages towards an M2 phenotype can enhance therapeutic effects of anti‐PD‐1 mAb against B16F10 melanoma, highlighting the crucial role played by TAMs in ICB resistance [63]. Thus, targeting the immunosuppressive cells may represent a pivotal strategy for overcoming drug resistance in AM.

TME harbors a diverse array of soluble immunosuppressive factors, including cytokines, metabolites, exosomes, and non‐coding RNA secreted by tumor cells, immune cells, or stromal cells. These components intricately interact to form a complex regulatory network that enhances immune suppression and facilitates evasion of immune surveillance by tumors. Transforming growth factor‐beta (TGF‐β) plays a dual role in regulating the interplay between tumors and the immune system. In the early stages of melanoma, TGF‐β inhibited tumor proliferation; while the TME of advanced melanomas was characterized by excessive secretion of TGF‐β established an optimal environment for tumor growth. Furthermore, TGF‐β might induce downregulation of MHC‐I expression ‐a pivotal marker of anti‐PD‐1 resistance [64]. However, TGF‐β signaling activation in AM cells indicates a favorable prognosis. These results illustrate entirely distinct roles for TGF‐β playing in treating different stages or subtypes of melanoma, requiring further validation for its influence on immune‐resistant AM [13]. CD73, known as a nucleotidase, catalyzes the hydrolysis of extracellular AMP into intracellular adenosine, which plays a promoting role in an inhibitory TME. Turiello R et al. found the activity of serum CD73 is lower in patients achieving complete or partial response compared to those with progressive disease (PD) after anti‐PD‐1 therapy [65]. In Chinese melanoma patients, AM was reported to account for a relatively large proportion, the expression of CD73 was more prevalent in tumor cells compared to PD‐L1 (54.6% vs. 23.2%). Moreover, an increase in CD73 expression was observed in 54.4% of PD‐L1 negative samples, suggesting its potential as an immunotherapeutic target next to PD‐L1 for AM patients [66].

Chemokines are small molecules produced by antigen‐presenting cells (APCs) or tumor cells to facilitate the recruitment of circulating cells to the tumor site, thus chemokines make a critical difference on modulating responsiveness to ICB therapy. Studies found the occurrence of CXCL9 and CXCL10‐rich milieus exhibited high expression of markers related to T cell dysfunction and exhaustion in the TME of melanoma [67]. Moreover, chemokines also impact the trafficking of immunosuppressive cells. Melanoma‐secreted CCL5 can attract MDSCs to the tumor while CCL22, CCL21, and CCL1 secreted by tumors attract Tregs to the TME in melanoma. These alterations shift the host immune response from immunogenic to tolerogenic, resulting in a low response rate to ICIs [68]. Some metabolites from the microenvironment also have a certain impact on immune resistance. The expression of Indoleamine 2,3‐dioxygenase 1 (IDO1), a crucial enzyme responsible for converting tryptophan (Trp) to uridine dinucleotide (Kyn), is significantly upregulated in melanoma cells following immunotherapy [69]. IDO1 could induce the development of an immunosuppressive TME of AM, suppress the activity of CD8+ T cells, NK cells, and DCs, as well as enhance the function of Tregs and MDSCs. Moreover, Li et al. discovered metastatic AM exhibited increased expression levels of metabolically relevant genes such as IGFBP4 and SLC25A4 compared to primary tumors [14]. Namiki, T. et al. found the risk of relapse was higher in AM patients with high expression of NUAK2 which functions as a sensor in cellular metabolism [70]. These results pave a new way for reversing the immunosuppressive TME in immune‐resistant AM patients through targeting chemokines or metabolism regulators.

#### 3.2.4 Other Factors

**FIGURE 2:**
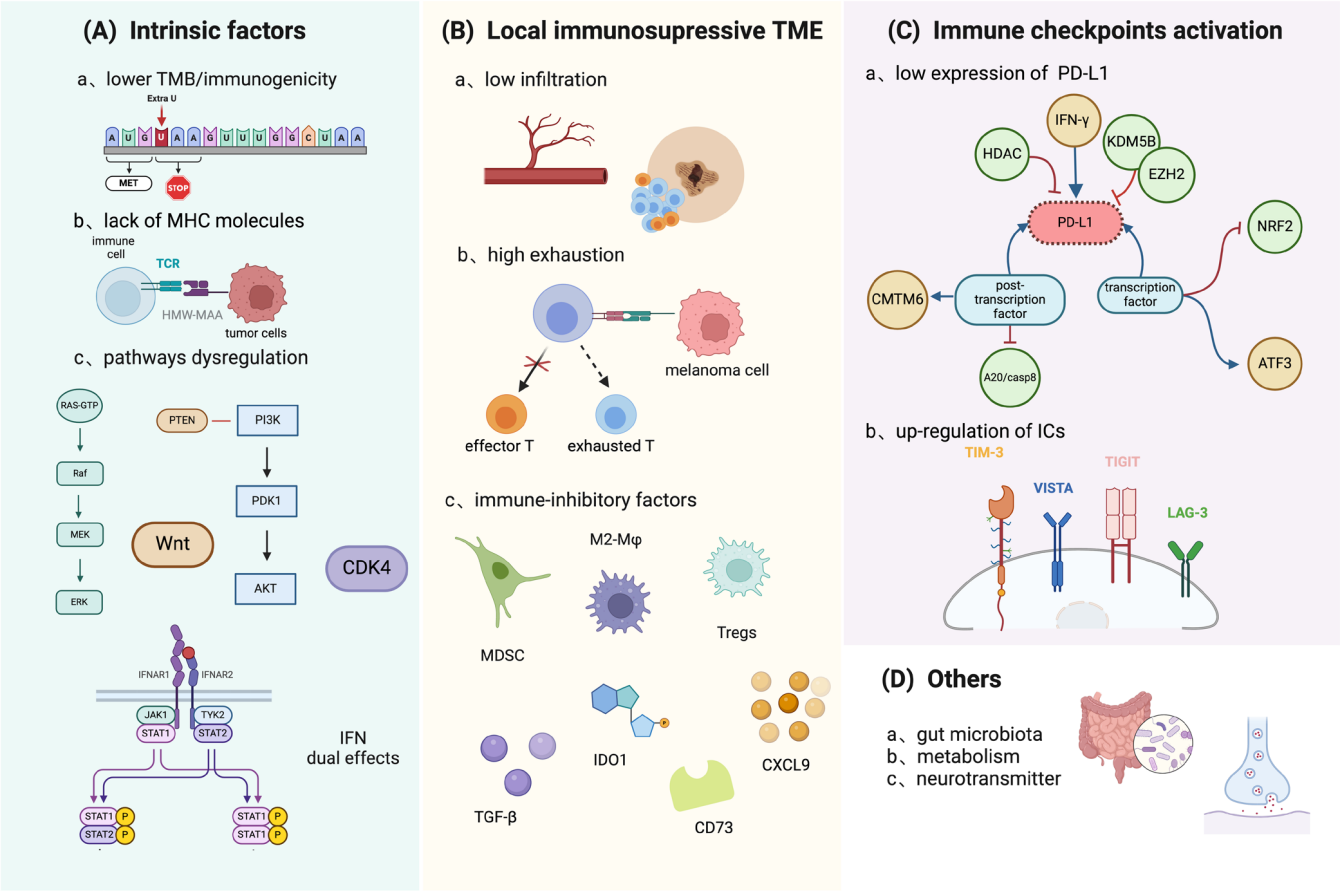
Potential mechanisms of immune resistance in AM. AM patients tend to develop immune resistance due to its unique immune TME. Three main possibilities are the cause of poor efficacy and temporary control for AM patients when treated with ICIs. (A) As for intrinsic factors, lower TMB indicates reduced expression of neoantigens while a lower expression of the membrane bound HMW‐MAA impairs antigen presentation. The alterations of signaling pathways also affect the recruitment, activation, and apoptosis of immune cells in AM, especially the WNT and CDK4 pathway. (B) The TME of AM exhibits a deficiency in effector CD8+ T cells while enrichment of exhausted CD8+ T cells with significantly reduced ability to kill tumor cells. Immunoregulatory cells such as Tregs, MDSCs, M2‐macrophages, and immunosuppressive factors such as TGF‐β, CD73, IDO1, and chemokines also increase in the TME, which leads to a reduction of ICIs efficacy. (C) PD‐L1, a classical inhibitory immune checkpoint that expresses lower in AM cells, is always associated with a poor prognosis and limited response to immunotherapy. Epigenetic modifications represent a useful method to regulate its expression. The inhibition of PD‐1 or CTLA‐4 potentially triggers the activation of other immunoregulatory pathways, resulting in a failure of current treatments. This figure was created with BioRender.com. AM, acral melanoma; HMW‐MAA, high molecular weight melanoma associated antigen; ICIs, immune checkpoints inhibitors; IDO1, indoleamine 2,3‐dioxygenase 1; MDSCs, marrow‐derived suppressor cells; TGF‐β, transforming growth factor‐beta; TMB, tumor mutation burden; TME, tumor microenvironment; Tregs, regulatory T cells.

In addition to the aforementioned factors, host‐related variables such as age and gender can exert an influence on the efficacy of immunotherapy [71]. Moreover, oral and gut microbiota play a regulatory role in enhancing anti‐PD‐1 therapy by upregulating immune cells like CD8+ T cells and antigen presenting molecules. Furthermore, increasing dietary fiber intake may improve treatment outcomes in immunologically tolerant patients with advanced melanoma through modulation of gut microbiota [72]. Metabolic changes in melanoma can impact the efficacy of ICB by regulating the immune characteristics of TME. Several studies have identified enhanced tumor cell glycolysis and oxidative phosphorylation that are correlated with ICB resistance, while amino acid metabolism and autophagy contribute to immune evasion in melanoma [69]. Melanomas also exhibit substantial metabolic heterogeneity; thus, it is crucial to analyze metabolic markers prior to initiating treatment and understand how metabolic dysregulation impacts resistance to immunotherapy. β‐adrenergic receptor (β‐AR)‐mediated signaling pathways have been proved to undermine ICB therapy through suppressing an effector phenotype in CD8+ T cells. Further research found β‐AR inhibitors can promote the transformation of melanoma into an immune‐active TME, representing a novel treatment strategy for using this approach during melanoma immunotherapy [73]. The potential mechanisms of immune resistance in AM are illustrated in Figure 2.

## 4 Systemic Therapy Combined With Immunotherapy

### 4.1 Current Combination Treatment Regimens

With lower response rate to immunotherapy and higher acquired drug resistance rate during the treatment, achieving the desired therapeutic effect in AM patients using a single immune or targeted therapy alone poses significant challenges in current clinical practice. Therefore, combining multiple therapies represents the most direct and advantageous approach for improving survival outcomes. Importantly, there is a pressing need to identify an optimal regimen that can mitigate the emergence of ICB resistance. The current combination therapies for AM are summarized in Table 1.

**TABLE 1:** Current combination treatments for AM patients.

Regimen	Clinical trial	AM (*n*)	ORR/DCR (%)	mPFS (month)	mOS (month)	Grade ≥ 3 irAEs (%)	Trial ID
**ICIs combined with chemotherapy**							
2019	Anti‐PD‐1 + temozolomide	13	38.5/61.5	UK	UK	UK	ACTRN12618000053224
**ICIs combined with anti‐VEGF agents**							
2023	Camrelizumab + famitinib	9	33.3	6	NR	UK	NCT05051865
2022	Camrelizumab + apatinib	30	24.1/82.8	7.39	13.4	50.5	NCT03955354
2022	Camrelizumab + apatinib+TMZ	50	64/88	18.4	UK	UK	NCT04397770
**ICIs combined with targeted drugs (dual)**							
2018	Atezolizumab + vemurafenib+cobimetinib	256	UK	15.1	UK	UK	NCT02908672
2019	Pembrolizumab + dabrafenib + trametinib	60	UK	16	UK	89.9	NCT02130466
2020	Spartalizumab + dabrafenib + trametinib	267	69	16.2	UK	55	NCT02967692
**Dual immune checkpoints inhibitors**							
*Anti‐PD‐1 + anti‐CTLA‐4*							
2014	Nivolumab + ipilimumab	314	UK	11.5	NR	59	NCT01844505
2015	Pembrolizumab + ipilimumab	153	61/79	NR	NR	UK	NCT02089685
2020	Anti‐PD‐1 + anti‐CTLA‐4	254	40	6.6	43.6	53	NN
	PSM	32	31	3.2	NR	UK	
	NAM	13	61	8.4	23.1	UK	
2022	Nivolumab/pembrolizumab + ipilimumab	59	43	5.4	15.6	35.6	NN
2020	Anti‐PD‐1 + anti‐LAG‐3						
	Nivolumab + relatlimab	82	43	10.1	19.3	18.9	NCT03470922
	Nivolumab + relatlimab	30	57	UK	UK	26	NCT02519322
	Cemiplimab + fianlimab	UK	63.8	UK	UK	UK	NCT03005782
	*Superior to any treatments	23	60.9	13.3	UK	43.9	
**Other combination**							
2022	anti‐PD‐1 + FMT	20	65	NR	NR (16 alive)	20	NCT03772899

Abbreviations: DCR, disease control rate; FMT, fecal microbial transplantation; irAEs, immune‐related adverse events; mOS, median overall survival; mPFS, median progression‐free survival; NAM, nail apparatus melanoma; NN, not numbered; NR, not reached; ORR, objective response rate; PSM, palm and sole melanoma; TMZ, temozolomide; UK, unknown.*This regimen demonstrates superior efficacy among the current combination regimens of dual immune checkpoint inhibitors for the treatment of acral melanoma.

### 4.2 ICIs Combined With Chemotherapy

Chemotherapy drugs represent classic utilized therapeutic approaches in cancer treatment. The combined application of chemotherapy and ICIs demonstrates a synergistic effect whereby chemotherapy alleviates tumor burden thereby promoting immunosurveillance for efficient elimination of malignant cells. Several studies demonstrated chemotherapy drugs have great potential to restore the sensitivity to immunotherapy. Treatment with azacytidine and carboplatin for ICB‐resistant metastatic melanoma significantly prolonged OS and PFS, while carboplatin and paclitaxel combination therapy for nivolumab‐resistant AM also conferred greater ORR and PFS than ipilimumab [74]. Surprisingly, a combination of anti‐PD‐1 mAb and temozolomide (TMZ) exhibits a remarkable outcome for AM patients, with higher ORR and longer duration of response (DOR) (38.5% and 11.1 months, respectively) than other types of melanomas [75]. Thus, these regimens deserve to be further assessed in clinical practice, particularly large, prospective randomized controlled studies for AM patients.

### 4.3 ICIs Combined With Anti‐Angiogenic Inhibitors

Anti‐angiogenic drugs targeting the VEGF pathway primarily exert their antitumor effects by counteracting VEGF‐induced immunosuppression, normalizing tumor vascular structure, and remodeling the TME. Combining these drugs with ICB therapy not only enhances immune cell infiltration at the tumor site but also blocks the immune inhibitory signals, thereby synergistically improving therapeutic outcomes and minimizing adverse reactions. Clinical evidence has demonstrated that combining anti‐PD‐1 mAb with anti‐VEGF agents, such as apatinib, famitinib, and axitinib, significantly improves the prognosis of patients with melanoma. The combination of camrelizumab and apatinib exhibited notable antitumor activity in advanced AM patients confirmed by a clinical trial at Peking University Cancer Hospital [76]. Building upon these two drugs, a triple regimen combined with TMZ has emerged as the most effective first‐line treatment for advanced AM, which obtained a satisfactory result with greatly improved ORR and PFS compared with placebo group [77]. However, the combination of camrelizumab and famitinib showed limited effects in treating AM. Currently, different subtypes of advanced melanoma exhibit varying degrees of clinical benefit and safety when treated using different combinations of anti‐VEGF drugs and ICIs. A combination of toripalimab and axitinib displays promising antitumor activity in patients with metastatic MM and may potentially impact the antitumor effect in AM patients [78]. However, further verification is required to determine the specific drug combinations and measurement schemes.

### 4.4 ICIs Combined With Targeted Drugs

The combination of targeted therapy and immunotherapy has shown rapid and long‐lasting effectiveness, helping to reduce the occurrence of drug resistance. Inhibition of BRAF and MEK can directly enhance the presentation of tumor antigens or increase the immunogenicity of tumor cells; simultaneously, it reduces the expression or release of immunosuppressive molecules while promoting infiltration by immune cells and activation of T cells. The combination of PD‐(L)1 blockade with BRAF inhibitors (vemurafenib, dabrafenib) and MEK inhibitors (trametinib, cobimetinib) shows promising potential in addressing drug resistance in advanced melanoma. Several clinical trials found the addition of a PD‐(L)1 mAb to dual targeted agents did not result in an improved ORR compared to placebo, but it significantly prolonged the DOR and PFS. The benefit group was primarily melanoma patients with high tumor load and elevated lactate dehydrogenase (LDH) [79, 80]. Furthermore, the sequence of therapy also makes some difference on the ultimate effects. According to retrospective research, a higher survival rate was observed in melanoma patients started with ICIs followed by BRAF and MEK inhibitors than those treated in the reverse order, indicating this therapeutic sequence should be preferred. Tatsuhiko Mori et al. examined the effectiveness of rescue therapy following anti‐PD‐1 mAb monotherapy failure in AM patients, demonstrating that BRAF and MEK inhibition can achieve superior ORR and PFS compared to other salvage therapies [81]. At present, there is limited clinical experience regarding the combination of ICIs with the BRAF/MEK inhibitors in treating AM patients, necessitating more clinical trials to determine an optimal triple regimen of this combination.

### 4.5 Immune Checkpoint Inhibitor Combination

The concept of dual immunotherapy is based on the application of PD‐(L)1 inhibitors, in conjunction with other ICIs, to further ameliorate the suppressive state within the TME. Numerous clinical trials have demonstrated that compared to monotherapy, dual immunotherapy can significantly augment recurrence‐free survival (RFS) in melanoma patients' postsurgery, particularly those exhibiting low PD‐L1 expression levels like AM. However, it should be noted that this approach has resulted in a substantial escalation in immune‐related toxicity, necessitating greater emphasis on drug tolerance and continuous administration.

In recent years, the combination of anti‐CTLA‐4 and anti‐PD‐1 mAb has emerged as the preferred regimen due to its capacity to restore T cell response against tumors during both priming and effector stages. Based on a 10‐year large‐scale prospective study (CheckMate 067), a significant improvement in OS and PFS was both present in the combination group (nivolumab & ipilimumab) compared to the single‐agent groups of AM, accompanied by enhanced drug tolerance and lessened adverse events [82]. For advanced AM, several retrospective cohort studies have shown the combination of anti‐PD‐1 mAb and ipilimumab leads to higher ORR and PFS compared to either anti‐PD‐1 mAb or ipilimumab alone, while there is no significant difference in OS [20]. Interestingly, this dual immunotherapy appears to be more efficacious in terms of tumor response for advanced nail apparatus melanoma (NAM), as opposed to palm and sole melanoma (PSM) [83].

Currently, novel dual immunotherapy approaches have presented promising possibilities for melanoma. Relatlimab, a LAG‐3 mAb, has demonstrated superior clinical efficacy when combined with nivolumab compared to single immunotherapy, while also exhibiting better safety than previous dual immunotherapies. When this regimen was applied in the neoadjuvant treatment of patients with advanced melanoma, high pathological response rates (pCR) were found to be associated with increased immune cell infiltration and reduced M2 macrophage infiltration at baseline [84]. As for other anti‐LAG‐3 agents, fianlimab used in combination with cemiplimab, showed significant clinical activity and the safety similar to that of cemiplimab alone in AM patients; however, LBL‐007 has not yielded substantial advantages in treating advanced melanoma. Additionally, sabatolimab and spartalizumab are mAbs that bind TIM‐3 and PD‐1, respectively. The combination of these two drugs was well tolerated and showed preliminary signs of antitumor activity in patients with advanced solid tumors including melanoma [85]. Consequently, compared to traditional dual immunotherapy, novel dual immunotherapies may represent an effective treatment approach with enhanced sensitization and lengthening survival periods, thereby holding significant clinical application value for AM in the future.

### 4.6 Other Regimens

Accumulating evidence from clinical trials has found that patients who received broad‐spectrum antibiotics prior to immunotherapy experienced a significant reduction of therapy response and survival, which can be explained by changes of the gut microbiota. Fecal microbiome transplantation (FMT) was proved to enhance the efficacy of immunotherapy by modulating gut microbiome composition and remodeling the TME of melanoma [86]. Simultaneously, in responders, FMT altered serum metabolome and downregulated multiple circulating cytokines associated with anti‐PD‐1 resistance [87]. These results indicate that FMT from healthy donors is clinically feasible for the first‐line therapy. However, there are still some issues regarding determining the most suitable donor, optimal dosage of FMT timing and method, evaluating need for FMT, etc., which require further exploration through randomized trials [88]. Meanwhile, it is unknown whether this regimen can also overcome the immune resistance of AM patients.

## 5 Promising Future Systemic Therapies for AM

### 5.1 Promote Immune Activation and Enhance Tumor Immunogenicity or Antigen Presentation

Oncolytic viruses possess the capacity to directly eliminate tumor cells and release tumor antigens, thereby facilitating the conversion of cold (immune tolerant) tumors into hot (immune sensitive) tumors. Relevant studies have demonstrated that combining T‐VEC (secreting human GM‐CSF) with anti‐PD‐1 therapy made a sustained response in the treatment of advanced melanoma (mostly PD‐L1‐negative) without any additional safety concerns. However, this approach did not result in significant improvements in PFS or OS [89]. Moreover, intra‐tumoral injection of an oncolytic virus engineered to coexpress a PD‐L1 inhibitor and GM‐CSF successfully overcame PD‐L1‐mediated immunosuppression in a B16‐F10 melanoma syngeneic mouse model, simultaneously leading to an effective rejection of both virus‐injected and distant tumors. Clinical trials that investigate the efficacy and safety of various oncolytic viruses combined with anti‐PD‐1 mAbs (BO‐112 plus pembrolizumab, OrienX010 plus toripalimab) are being conducted in the AM patients [90]. However, the number of patients recruited is too low to draw a comprehensive conclusion about the responses. So, a larger‐scale study is needed to investigate the safety, clinical activity, and pharmacodynamics of this new combination for AM.

The tumor neoantigen vaccines represent an innovative medical technology aimed at utilizing specific tumor antigens to elicit a targeted immune response against cancer cells. The specific neoantigens with optimal potential to activate antitumor immune responses were identified and encoded by gene sequencing analysis on tumor tissues. Recently, mRNA‐4157, a melanoma vaccine that heralds a new era in personalized tumor neoantigen therapy, effectively reduced recurrence risk (44%) when combined with pembrolizumab over anti‐PD‐1 mAb alone for high‐risk melanoma patients [91]. Another personalized vaccine (NEO‐PV‐01) and nivolumab combination in patients with advanced melanoma also proved to be feasible and safe, which even detected epitope spread to neoantigens not included in the vaccine postvaccination. Obviously, the auspicious application prospects of this technology are now explicit, but it is necessary to further understand the optimal injection time and dose of vaccines and whether this therapeutic regimen could make some difference on the survival of AM.

The efficacy of radiotherapy is intricately linked to the immune status and local immune microenvironment of the body. Radiotherapy‐induced immune activation enhances the likelihood of tumor cells being recognized by the host immune system; radiation concurrently shapes diverse immune TME with distinct activation patterns in tumor cells, stromal cells, and immune cells, ultimately facilitating recruitment and infiltration of immune cells. For metastatic melanoma, the utilization of ICIs provides an opportunity to enhance radioimmune stimulation potential, which isembodied in the increased ICOS+ T cells (immune‐activated) presenting in the combination groups with ipilimumab and radiotherapy [92]. The combination of local radiotherapy and immunotherapy has also exhibited the effectiveness and safety in the treatment of AM in situ, particularly by improving OS. However, a Japanese study suggested that the clinical response in the combination of radiotherapy and anti‐PD‐1 therapy could be less effective in AM than mucosal melanoma (MM) patients [93]. Simultaneously, it is crucial to consider synergistic toxicities and a heightened risk of adverse events between these high toxic treatments.

Hyperthermia is a widely utilized therapeutic modality in localized ablation for cancer treatment. Hyperthermia primarily enhances antitumor immunity by inducing “immunogenic cell death” (ICD). This process releases danger signals (calreticulin, HMGB1, ATP) and heat shock proteins, which efficiently activate dendritic cells and T cells to initiate a specific immune response. Numerous studies have demonstrated that hyperthermia therapy can induce a highly immunogenic TME of melanoma, thereby simultaneously eliminating primary tumors and metastatic lesions, even leading to the prevention of tumor recurrence. In the xenograft model for B16F10 cells, several studies found that the combination of SMPAI and anti‐PD‐1 mAbs prolonged the life span of mice, implying the increase of intra‐tumoral APCs and CD8^+^ T cells [94]. Additionally, following cellular injury posthyperthermia, protective upregulation of inhibitory molecules such as PD‐L1 occurs to prevent excessive immune activation, indicating it may make an improvement on the sensitivity to ICB therapy especially for AM (lower PD‐L1 expression) [95]. Consequently, although combining ICIs with hyperthermia has not been widely applied in melanoma, this regimen has tremendous potential for treating immune‐resistant AM due to its high capabilities of immune activation and low poisonousness.

Toll‐like receptor 9 (TLR9) is an intracellular receptor that initiates innate immune responses and can be modulated or methylated in human cells. Vidutolimod, a virus‐like particle containing a CpG‐A TLR9 agonist, elicits robust IFN production, thereby inducing the recruitment of antitumor T cells and overcoming resistance to anti‐PD‐1 mAbs. Clinical studies have demonstrated the significant capacity of vidutolimod administration to induce tumor regression when combined with pembrolizumab in patients with PD‐1‐refractory melanoma. Furthermore, a combination with tilsotolimod (another TLR9 agonist) and ipilimumab similarly resulted in promising prognosis in patients with melanoma by enhancing antigen presentation and improving tumor immunogenicity through up‐regulation of MHC2 molecules and PD‐L1, respectively [96, 97]. Therefore, this therapeutic regimen could be considered as a viable or backline option for reversing immune resistance in AM.

### 5.2 Promote the Activation of Immune Cells in TME and Intra‐Tumoral Movement

The stimulator of interferon genes (STING), an intracellular receptor, plays a crucial role in regulating innate immunity. Upon DNA damage in tumor cells, stimuli are released that activate the STING protein which initiates cellular senescence and release of inflammatory mediators and cytokines as well facilitates DC maturation and recruitment of supportive immune cells, thereby augmenting the antitumor response rate of immunotherapy. Combining STING agonists with ICIs has exhibited significantly enhanced antitumor activity. These regimens led to greater outcomes of anti‐PD‐1‐refractory melanoma patients compared to other tumor types, with the same efficacy between high‐frequency and low‐frequency groups [98]. SYNB1891 is a nonlethal strain of *E. coli* that produces cyclic dinucleotides under hypoxia and activates STING in phagocytic APCs within tumors. Repeated injections of SYNB1891 for patients with advanced/metastatic melanoma as monotherapy and in combination with atezolizumab (a PD‐L1 inhibitor) both exhibited favorable safety profiles and tolerability [99]. However, several problems remain to be solved, such as a rapid drop in plasma concentrations and deleterious changes to effector T cells after using STING agonists.

CAR‐T cell therapy has demonstrated promising clinical outcomes in hematologic malignancies; however, the efficacy analysis in solid tumors reveals an only 9% comprehensive response rate. The primary limiting factors encompass the difficulty in trafficking of CAR‐T cells into the tumor site, as well as the prolonged antigen stimulation leading to exhaustion phenomena within CAR‐T cells. Some studies have demonstrated that the expression of immune checkpoint molecules, namely PD‐1 and LAG‐3, is upregulated after the activation of certain CAR‐T cells in metastatic melanoma. Consequently, combining ICIs with CAR‐T cells can overcome the inhibitory effects imposed by the TME and has exhibited initial success in treating hematological tumors [100]. The sustained efficacy of TILs therapy following failure of ICB has also been confirmed. Notably, melanoma patients who were refractory to anti‐PD‐1/PD‐L1 mAbs all achieved favorable treatment responses upon receiving Lifileucel (an autologous TILs) [101]. For metastatic melanoma patients who received TILs with nivolumab, a majority of whom achieved complete adoptive cell transfer (ACT) (9/11), and CD8+ TILs were successfully expanded from all patients and were tumor reactive in vitro. However, patients who previously received frontline ICB followed by TILs therapy exhibited diminished expansion compared to their initial treatment, and there still exist dysfunctional T cells within the tumors [102]. These findings collectively suggest that early combination therapy with ACT therapy and ICIs could be considered as a viable option for AM patients, which merits more proof‐of‐concept clinical trials and basic research.

Cytokines play a pivotal role in the initiation, progression, and eradication of cancer, as well as in regulating various facets of innate and adaptive immunity. Research has demonstrated that combining cytokines with ICIs can generate synergistic antitumor effects. By leveraging the immunomodulatory properties of IL‐12, intra‐tumoral injection of TAVO (an IL‐12 plasmid) can precisely ameliorate primary drug resistance caused by loss of the IL‐12/IFNγ pathway in melanoma patients. Furthermore, combining TAVO with pembrolizumab for advanced melanoma patients exhibiting low checkpoint positive TILs infiltration within tumor yielded favorable results compared to TAVO monotherapy (ORR 41% vs. 35.7%), underscoring its potential for achieving improved efficacy in AM patients [103]. Interferon (IFN) is a classic agent in oncotherapy, which has been applied for decades but yielded limited clinical benefits and the application of IFN‐α 2b was limited by its severe toxicity. Notably, the combination of IFN‐α 1b with anti‐PD‐1 mAb was active and well tolerated for melanoma patients in China, especially for AM that showed an above average ORR in all patients [104]. In addition, the combination of CD122‐biased agonist (NKTR‐214) that activates IL2 pathway preferentially and nivolumab has been proved to enhance efficacy through the activation of immune cells in TME for melanoma. Posttreatment analysis of the microenvironment revealed a significant increase in NK cells and CD8+ T cells within the tumor, as well as a conversion of 53% of tumors from PD‐L1 negative to positive status, indicating NKTR‐214 may become a promising drug coadministered with ICIs for treating AM patients [105]. However, there are less biomarkers available to predict the responses and side effects of cytokines, which may restrict their application in high‐risk patients. Meanwhile, the clinical application of cytokines is always limited due to their pleiotropic effects, short half‐life, and extensive toxicity profiles. Thus, it is important to monitor the changes in cytokine levels during the treatment.

The suspension of heat‐inactivated whole‐cell mycobacteria (IMM‐101) has exhibited efficacy in activating diverse innate immune cells and demonstrates notable antitumor activity in vivo. Dalgleish AG et al. unveiled that coadministration of ICIs and IMM‐101 yielded a high response rate rapidly and was devoid of any additional toxicities among treatment‐naive patients with advanced melanoma. However, subsequent disease progression was observed among immune‐refractory patients, indicating this regimen may also drive the immune response disadvantageously [106]. Notably, an enrolled AM patient who received thereafter ipilimumab after IMM‐101 experienced a rapid response with most of the lesions resolving and a couple remaining stable over a long period of time [107]. Therefore, more trials are warranted to evaluate the potency in treating AM patients with the combination of bacteria and ICIs.

### 5.3 Reverse the Immunosuppressive State of TME

Drug resistance is frequently associated with an inhibitory TME, underscoring the importance of targeting the factors that contribute to immunosuppression. Consequently, drugs aimed at modulating immunosuppressive factors have gained traction in various immune combination therapies.

The blockade of IL‐6 that mainly functions as an immune‐suppressive cytokine, is supposed to potentiate antitumor immunity, and mitigate the toxicity induced by ICIs. Nevertheless, IL‐6R inhibitors (tocilizumab/sarilumab) have exhibited a remarkable reduction of immune‐related adverse events (irAEs) while shown minimal influence on the effectiveness of ICB therapy in AM patients [108]. When combining TNF inhibitors (certolizumab/infliximab) alongside ipilimumab and nivolumab for treating metastatic melanoma, a boost in ICB efficacy was observed without notable toxic effects [109]. Furthermore, cytokine release syndrome (CRS), a severe immune‐ related complication caused by ICIs, could also be avoided by specific agents targeting the elevated cytokines.

**FIGURE 3:**
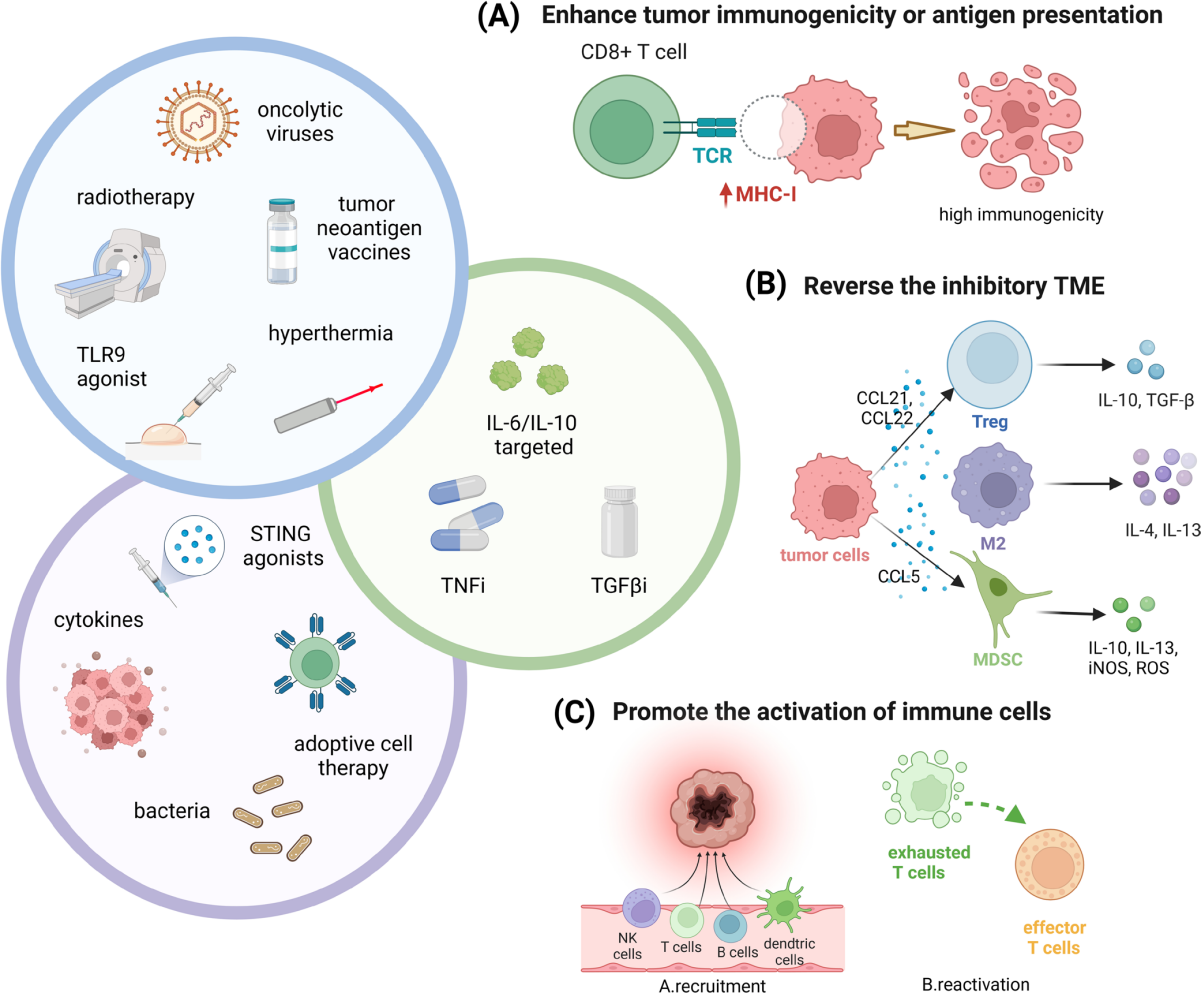
Promising future systemic therapies for AM patients. Based on the current understanding of characteristics and drug resistance mechanisms of AM, we summarize existing key strategies combined with ICIs and other agents to improve therapeutic outcomes without adding extra toxicity. (A) Enhancing tumor immunogenicity or antigen presentation can promote the conversion of cold tumors into hot tumors. Through upregulation of the MHC molecules in melanoma cells or releasing tumor neoantigens, oncolytic viruses, mRNA vaccines, radiotherapy, hyperthermia, and TLR9 agonists have great potency to reverse immune resistance. (B) Reversing the immunosuppressive state of TME needs to decrease the infiltration of Tregs, M2‐mmacrophages, MDSCs within the tumors or eliminate inhibitory molecules such as IL‐10, TGF‐β, IL‐13. Thus, the blockade of IL‐6, IL‐10, TGF‐β and TNF may restore the activation state of TME. (C) Promoting the activation of immune cells in TME not only facilitates the maturation and movement of supportive immune cells such as T cells, B cells, NK cells and DCs, but also sparks a conversion of immune cells from exhausted to effector state. STING agonists, adoptive cell therapy, cytokines and special bacteria preliminarily ameliorate immune resistance and are warranted further evaluated. This figure was created with BioRender.com. AM, acral melanoma; ICIs, immune checkpoints inhibitors; STING, stimulator of interferon genes; TCR, T cell receptor; TGF‐β, transforming growth factor‐beta; TLR9, toll‐like receptor 9; TME, tumor microenvironment.

However, some cytokine‐targeted therapies have faced sluggish progress due to their dual impact on patient survival. The suboptimal efficacy of TGF‐β inhibitors combined with ICIs may be explained by the proliferation of stromal fibroblasts, which can develop drug resistance through MMP9‐dependent cleavage of PD‐L1 protein. Nevertheless, delayed TGF‐β inhibitors therapy may better be served to control further disease progression compared with a continuous combination. Selective TGF‐β inhibitors secreted by Tregs also exhibited potent ability to overcome drug resistance by increasing antitumor activity [110]. IL‐10, an inhibitory cytokine, has been proved to facilitate the invasion and metastasis of melanoma. Furthermore, it was found to be highly expressed in the immune cells of AM, indicating a prospective target to directly restore the immune activation and specifically change the immune TME in AM [111]. Consequently, reactivating the immune cells within the TME necessitates the identification of novel targets that highly correlated with immunosuppression and specifically expressed in AM. Promising future systemic therapies for AM patients are summarized in Figure 3.

## 6 Current Challenges in the Immune Therapy of AM

AM, a subtype of melanoma, exhibits a high incidence rate and accounts for nearly 50% of cases in non‐Caucasian populations. It is characterized by difficulties in detection, rapid progression, poor prognosis, and many challenges in treatments. First, patients face limited treatment options with low response rates to current therapies and an increased susceptibility to acquired immune resistance. Although some combination regimens have achieved a better ORR than single agents, they did not avoid short‐term recurrence or rapid progression of the disease, indicating novel treatments that can simultaneously achieve high ORR and PFS in AM patients are still not available [89]. PD and irAEs are the most common reasons for therapy to be discontinued. Secondly, most AM patients treated with ICIs will experience irAEs, especially in the circumstance using multiple targeted or immune drug treatment. It needs to be noticed that high‐grade irAEs and high‐risk immune‐related complications such as CRS may directly endanger the patients' lives [112]. Thirdly, although the therapeutic effects of current treatments are not ideal for AM, more targeted treatment is still scarce and it did not attract enough attention becauseAM is a rare type in Caucasians [113]. Therefore, it is imperative to explore effective approaches for addressing drug resistance through innovative treatment modalities or combination therapies.

As for the ongoing clinical trials on AM, only limited patients were enrolled and most patients were at an early stage. Moreover, single‐arm or single‐center retrospective trials that account for most of current clinical tests make the results less convincing. Therefore, large‐scale, prospective trials are urgently needed, which will be performed by different groups to compare efficacy and safety of new combination strategies applied on the patients of AM and CM. In the meantime, AM animal models such as mouse xenograft tumors and more specific biomarkers to predict the efficacy of immunotherapies should be paid much attention in the future investigations.

## 7 Conclusions and Future Perspectives

Immunotherapy represents a pivotal approach in the management of advanced cancers, effectively delaying cancer progression and enhancing patients' expected survival time. However, immunotherapy alone often proves ineffective or has low efficacy in AM. Thus, the combination of immunotherapy with other treatment modalities has emerged as the prevailing trend in managing advanced or metastatic melanoma. Currently, diverse combination therapies aimed at overcoming ICB resistance have been in clinical trials, which encompass combinations of ICIs with anti‐angiogenic drugs, targeted agents, chemotherapy, or dual immune strategies. The integration of multiple approaches significantly improves patient response rates and restores an “activated” state to the immune microenvironment. To further augment antitumor immune responses, many regimens through different therapeutic strategies are being investigated, including enhancing tumor immunogenicity or antigen presentation, promoting the activation and infiltration of immune cells within the tumor, and reversing the immunosuppressive TME. It is noteworthy that the combination of immunotherapy with other therapies may also increase the incidence of adverse events, particularly immune‐related side effects such as pneumonia and CRS. Therefore, it is imperative to focus on controlling toxicity associated with combination therapy within safe limits so that patients can better tolerat the therapy and achieve long‐term survival improvements.

In this review, we summarize the latest clinical research data on immunotherapy for advanced melanoma, with a particular focus on AM. The combination of ICIs and targeted drugs or ICIs and anti‐VEGF agents showed a relatively higher ORR while triple‐therapy with ICIs, anti‐VEGF drug, and chemotherapy achieved the best PFS. Surprisingly, a new dual ICIs treatment including anti‐PD‐1 and anti‐LAG‐3 mAbs significantly prolonged the PFS of AM patients compared to single ICIs and exhibited lower immunotoxicity. However, there is no study with comprehensive clinical data on AM patients, and clinical trials of drugs specially for AM are very limited. So, evaluating the therapeutic effects of different drug combinations in AM systematically is hard work at present.

In addition, we provide insights for future combination strategies in refractory or drug‐resistant cases of advanced AM. Oncolytic viruses such as T‐VEC not only make higher response rate and extended survival without extra adverse events, but also demonstrate an effective rejection of both virus‐injected and distant tumors just like STING agonists. However, the STING agonist is less recommended for treating refractory AM patients due to its rapid drop in the concentrations of plasma and tumors after injection. Tumor neoantigen vaccines such as mRNA vaccines are also promising choices for combining treatments, because of a sharp decrease in the recurrence risk of high‐risk AM patients when treated with this combination regimen. To reduce the immune‐related toxicity caused by ICB therapy, some cytokine drugs such as IFN‐α 1b are often used to increase the tolerance against ICIs and simultaneously make clinical response more durable, indicating their well applicability for treatingAM patients with severe irAEs. The different systemic strategies for the combination treatment of AM are compared in Table 2.

**TABLE 2:** Compare different systemic strategies for the combination treatment of AM.

Methods/Drugs	Advantages	Disadvantages	Applicability
**Promote immune activation, enhance tumor immunogenicity or antigen presentation**			
*Oncolytics viruses*			
T‐VEC	Higher response rate without extra adverse events	No significant improvements of PFS or OS	Strongly recommend
sRP1	Extended survival compared with placebo group	Patients‐included is too low	
BO‐112	An effective rejection of both virus‐injected and distant tumors		
OrienX010			
*Tumor neoantigen vaccine*			
mRNA‐4157	High specificity and is independent of TMB	Large Individual differences	Recommend
NEO‐PV‐01	Induce epitope spreading; decrease the recurrent risk, especially for high‐risk patients	Uncertain of optimal injection time and dose; lack of clinical evidence	
Radiotherapy	Significantly increased infiltration of T cells	Limited efficacy while relatively toxic	Less recommend
*Hyperthermia*			
IR820	Precise localization minimizes damage to surrounding tissues; induce a highly immunogenic TME; elimate primary tumors and metastatic leisions	Minimal clinical trials; need to establish more complete temperature‐effect relationships	More trials to evaluate
*TIL‐9 agonist*			
Vidutolimod	Significant tumor regression at both injected sites and non‐injected components	Numbered choice of drugs	Recommend
Tilsotolimod	Rapidly induce and attract antitumor T cell	The optimal doses varied in the trials	
**Promote the activation of immune cells in TME and intratumoral invasion**			
*STING agonist*			
MIW815	Clinically feasible	Minimal efficacy in refractory patients	Less recommend
SYNB1891	Exist same efficacy between high‐frequency and low‐frequency group	Rapid drop in plasma concentrations (reflect quick clearance within tumor); deleterious changes to effector T cells at both lower/higher doses	
*ACT*			
CAR‐T	Achieve completed ACT*	Diminished expansion in resistant patients compared to ICB‐native	Recommend
TILs	Predominantly CD8+ TILs are successfully expanded	Dysfunction of infiltrated T cells (like low recognition of autologous tumor)	
*Cytokines*			
IL‐12 (TAVO)	Deep, durable and frequent clinical response	Pleiotropic effects, short half‐life, and extensive toxicity profiles	strongly recommend
IFN‐α 1b	Apply to patients with a low frequency of cpTILs in TME	Limited therapeutic spectrum of tumors (only superficial accessible)	
CD122 agonist	Lessen high rates of severe side effects caused by systemic administration	Less biomarkers predicting the responses	
*Mycobacteria*			
IMM‐101	A rapid response; absence of any severe systemic toxicity	Only small cohort studies; may also drive the immune response disadvantageously	More trials to evaluate
*Reverse the immunosuppressive state of TME*			
Anti‐IL‐6 (R)	Reduce irAEs (anti‐inflammatory)	Dual effects restrict its application	Recommend
TNF inhibitors	Avoid high‐risk immune‐ related complications (like cytokine release syndrome)		
TGF‐β inhibitors			

Abbreviations: ACT*, adoptive cell transfer; ACT, adoptive cell therapy; CAR‐T, chimeric antigen receptor T cell; cpTILs, checkpoint positive cytotoxic lymphocytes; ICB, immune checkpoints blockade; irAEs, immune‐related adverse events; ORR, objective response rate; OS, overall survival; PFS, progression‐free survival; TILs, tumor‐infiltrating lymphocytes; TMB, tumor mutation burden; TME, tumor microenvironment.

In the future, large‐scale, multicenter clinical trials are crucial for evaluating the efficacy, durability, and safety of ICIs combined with different treatment modalities in treating AM. To find a more precise target, it needs more basic and clinical studies focusing on the mechanisms of immune therapy resistance, especially those aiming at the characteristics of immune TME of AM. Moreover, individualized analysis of AM patients is necessary to find the most effective treatment regimens that substantially optimize survival outcomes and reduce the medical expenses. Consequently, it is still a long way to thoroughly recognize the occurrence and development of ICB resistance and select the optimal combination therapies for AM patients.

## Author Contributions

Lihong Jiang drafted the manuscript and counted and plotted the diagrams and tables. Zhaotian Zhang downloaded the data in public databases. Lihong Jiang and Zhaotian Zhang finished the collection and analysis of data. All authors read and approved the final manuscript.

## Funding

The authors have nothing to report.

## Ethics Statement

The authors have nothing to report.

## Consent

The authors have nothing to report.

## Conflicts of Interest

The authors declare no conflicts of interest.

## Data Availability

The raw bulk RNA sequencing data generated in this study comes from the Gene Expression Omnibus (GEO) database under accession code GSE215121 and GSE189889. The public data used in this study are available in the TCGA database.
